# Q-LEASE: Lifecycle-bound zero-trust authorization for HPC-to-QPU job handoffs

**DOI:** 10.12688/openreseurope.24782.1

**Published:** 2026-08-24

**Authors:** Fouad Ailabouni, Jesús-Ángel Román-Gallego

**Affiliations:** 1 Departamento de Informática y Automática, Escuela Politécnica Superior de Zamora, Universidad de Salamanca, Zamora, 49029, Spain; 2 CIMET Research Group, Universidad de Salamanca, Zamora, 49022, Spain; 3 AIR Institute – National Technological Center in Castilla y León, Salamanca, 37003, Spain; 4 BISITE Research Group, Universidad de Salamanca, Salamanca, 37007, Spain

**Keywords:** zero trust, quantum computing security, capability-based access control, proof of possession, formal verification, access control lifecycle

## Abstract

**Background:**

Zero-Trust access control for cloud APIs is well established, but hybrid High-Performance Computing (HPC)-to-Quantum Processing Unit (QPU) workflows have a lifecycle existing mechanisms were not built for: a job queues for minutes to days, is dispatched, executes, and only then releases results, while identity, policy, and hardware calibration state can change throughout. Existing token, proof-of-possession, and workload-identity mechanisms do not separate permission to queue from permission to execute, nor bind authorization to current hardware state.

**Methods:**

We designed and implemented Q-LEASE, a two-capability protocol separating a Queue Admission Ticket from a single-use Execution Capability bound to a circuit hash, backend identity, and calibration epoch, re-evaluated by a real Open Policy Agent (OPA) engine immediately before dispatch. The prototype uses real Ed25519/PASETO tokens, DPoP-style proof-of-possession, SPIFFE-shaped workload identity, and a phase-aware revocation state machine, deployed as six containerised services on a Kubernetes ( kind) cluster with Kueue-driven queueing. We compared Q-LEASE against four representative baselines across 16 attack/failure scenarios (2400 attack trials, 500 clean trials), and verified core safety properties with TLA+/TLC and token-flow security with the Tamarin prover.

**Results:**

Q-LEASE blocked 90.0% of unauthorized-execution attempts (95% confidence interval (CI) 87.0–92.4%), versus 12.5–37.9% for the four baselines. The remaining gap came from queue/execution separation and circuit/backend/calibration-epoch binding, which no baseline implements. Both TLA+ safety properties held, and all five Tamarin lemmas were proved automatically. Q-LEASE’s mean policy-decision latency was 44.3 ms, approximately 34.4 ms higher than the baselines, with no measurable increase in end-to-end workflow completion time relative to a real, contention-driven queue wait, while running against a real Kueue-admitted workload on a real cluster.

**Conclusions:**

Separating queue admission from execution authorization, and binding it to hardware state re-checked immediately before dispatch, closes a gap that composing existing OAuth-family mechanisms does not, at a latency cost small relative to realistic QPU queue waits.

## Plain Language summary

Quantum computers are rare and expensive, so most people who want to run a job on one first submit it from a regular supercomputer, then wait in a queue – sometimes for hours – before it actually runs. Today’s security mechanisms for this handoff mostly reuse ideas built for ordinary web logins: a temporary access card that is either too short-lived (so the job fails while still waiting) or too long-lived (so a stolen card stays dangerous for hours). We built and tested a different approach: split the “permission to wait in line” from the “permission to actually run the job” into two separate, narrower access cards, and only issue the second one at the last possible moment, after re-checking that nothing important has changed – who is asking, whether the hardware is still calibrated well enough, and whether the exact computation being requested still matches what was originally approved. We built a real, working version of this system, tried to break it with 16 different attacks, and compared it against four approaches that resemble what is used in practice today. Our system blocked 90% of attacks, versus 12–38% for the existing approaches, while adding only a small, measurable delay. We also used two independent mathematical verification tools to check that the core safety rules of our design cannot be violated, and both confirmed they hold.

## 1. Introduction

Hybrid classical–quantum workflows – variational eigensolvers, quantum-approximate optimisation, and circuit-cutting pipelines among them – increasingly submit quantum jobs from an HPC scheduler to a remote QPU provider as one step in a larger classical workflow. The Zero-Trust model formalised in National Institute of Standards and Technology (NIST) Special Publication (SP) 800–207 replaces implicit network trust with per-access policy decisions based on the subject, resource, and context of each request. ^1^ Applying Zero Trust to this handoff sounds like a natural extension, but the mechanisms available to do so were built for a different shape of workload than a queued quantum job.

OAuth Token Exchange lets an authorisation server exchange one security token for a narrower one in delegation workflows ^2^; DPoP binds an access token to a caller-held cryptographic key so that a stolen token alone is useless ^3^; and SPIFFE issues short-lived, verifiable workload identities that do not depend on network location. ^4^ These are implementation-level standards, distinct from the peer-reviewed research literature reviewed next, and we draw on them as building blocks rather than as prior contributions to displace.

Five bodies of peer-reviewed research bear on this gap, each addressing a necessary but insufficient part of it. *Continuous authorisation and usage control* established that an authorisation decision can remain active, and be revisited, after access begins, depending on mutable attributes, obligations, and environmental conditions, ^5^ with later work formalising usage-control policies and analysing their safety ^6^ ^,^ ^7^; continuous-authorisation frameworks and recent Zero-Trust dynamic access-control engines extend this principle operationally. ^8^ ^–^ ^11^ This establishes the general principle Q-LEASE relies on – that authorisation may be reassessed as state changes – but none of it defines a quantum-job lifecycle with separate authority for queue admission, dispatch, execution, and result release, nor binds a one-time execution authority to a circuit hash, a selected backend, and a hardware-calibration epoch. *Scoped capabilities for scientific computing* are likewise established practice: macaroons attach contextual caveats restricting where, when, and for what purpose a credential may be used, ^12^ and SciTokens, SciTokens SSH, and SciAuth apply capability-based authorisation to remote scientific data, high-energy-physics computing, and interactive login respectively. ^13^ ^–^ ^17^ Q-LEASE does not claim to invent scoped capabilities, short-lived credentials, or capability-based authorisation for scientific workflows; its contribution is narrower and domain-specific – two capability classes with disjoint authority, in which a Queue Admission Ticket can enqueue, poll, or cancel but never authorise execution, and a fresh Execution Capability is minted only at dispatch, after the job, policy, backend, and calibration state are rechecked. *Quantum-cloud security and trusted execution* protects the confidentiality and integrity of a delegated quantum computation once it reaches the provider: QEnclave-style secure delegated quantum computing ^18^ and quantum trusted-execution-environment architectures ^19^ ^–^ ^21^ protect a circuit and its control information from an untrusted cloud operator, while side-channel and crosstalk studies demonstrate concrete risks from shared control infrastructure and multi-tenant execution. ^22^ ^,^ ^23^ This motivates stronger protection for cloud quantum computing generally, but addresses secure execution and isolation once a job is already running, not who may move a queued job into execution after a possibly long delay or how authorisation should respond to revocation at different lifecycle phases. *Quantum scheduling and orchestration* addresses an adjacent systems problem: qubit-mapping and multi-programming mechanisms for shared QPU capacity, ^24^ ^–^ ^26^ hybrid quantum-classical workflow formalisation, ^27^ quantum operating-system and virtualisation abstractions, ^28^ ^,^ ^29^ cloud orchestrators and reinforcement-learning-based schedulers, ^30^ ^–^ ^33^ and distributed quantum-HPC workflow orchestration including circuit cutting. ^34^ This literature establishes that queueing, backend selection, calibration-aware placement, and hybrid orchestration are real, actively studied systems problems, but it addresses *where* and *when* a job should run, not a lifecycle-bound authorisation protocol in which queue admission is deliberately insufficient for execution and a separate, single-use capability must be minted after a dispatch-time policy decision. Finally, *calibration drift and dispatch-time freshness*: quantum processors exhibit measurable time-dependent drift, ^35^ and just-in-time transpilation has been proposed specifically to reduce the mismatch between compilation decisions and the hardware state at execution time. ^36^ This supports treating current backend state and calibration epoch as dispatch-time inputs rather than immutable submission-time facts, but neither paper frames calibration state as part of a cryptographically enforced authorisation binding.

Read together, this literature shows that continuous and usage-based authorisation, scoped scientific-computing capabilities, quantum trusted execution, quantum scheduling and orchestration, and calibration-aware compilation each exist as separate, independently developed research threads. To the best of our knowledge, none of them jointly defines and evaluates a lifecycle-specific protocol that separates queue-admission authority from execution authority, performs a fresh policy decision at dispatch time, binds a single-use execution capability to the exact circuit, backend, and calibration epoch, defines revocation semantics across queueing, execution, completion, and result release, and verifies these properties both formally and experimentally in an HPC-to-QPU workflow – an inference from the reviewed corpus, not a claim that no related implementation of any kind has ever existed elsewhere. We accordingly do not claim the first use of Zero Trust for quantum computing, the first short-lived or proof-of-possession-bound QPU credential, the first capability token for scientific computing, or that OAuth Token Exchange, DPoP, SPIFFE, macaroons, usage control, or SciTokens are insufficient in general: each supplies a useful building block, but their composition does not by itself impose Q-LEASE’s queue/execution separation, dispatch-time physical-state binding, or lifecycle-phase-specific revocation semantics.

This leaves a gap that is easy to misstate. The gap is *not* that quantum jobs need short-lived credentials – short-lived workload credentials are well understood. The gap is that existing identity, Zero-Trust, and quantum-orchestration mechanisms do not define lifecycle-aware authorisation semantics for a quantum job whose submission, queueing, compilation, dispatch, execution, and result release occur at different times, under changing hardware, policy, and workflow conditions. A token valid for the whole queue wait creates an unnecessarily large attack window; a token that expires before dispatch fails a legitimate job. Existing mechanisms give a designer the primitives to build a solution to this problem, but not the solution itself.

We aimed to design, implement, and adversarially evaluate a lifecycle-bound, two-capability authorisation protocol – separating permission to enter a QPU queue from permission to execute, and re-evaluating identity, workflow intent, data policy, and QPU state immediately before dispatch – and to determine, empirically and formally, how much of its protection existing OAuth-family mechanisms already provide versus how much comes specifically from this separation. Section 2 details six research questions this evaluation was designed to answer, covering the authorisation separation itself, dispatch-time reauthorisation overhead, which QPU-specific attributes should inform a Zero-Trust decision, phase-dependent revocation, resistance to capability substitution, and behaviour under long or unpredictable queue delay.

## 2. Methods

### 2.1 Study design

This was a systems-security design-and-evaluation study, structured around six research questions: (RQ1) how authorisation should be separated between queue admission, dispatch, execution, and result release; (RQ2) whether dispatch-time reauthorisation prevents stale or revoked jobs without materially increasing turnaround time; (RQ3) which QPU-specific attributes should inform a Zero-Trust decision; (RQ4) how revocation should behave when a job is queued, executing, or completed; (RQ5) whether capability-bound authorisation prevents circuit, backend, shot-budget, and result-destination substitution; and (RQ6) how the protocol behaves under long or unpredictable queue delay. We addressed RQ1–RQ4 through protocol design and formal verification, and RQ5–RQ6 through adversarial empirical evaluation against four baselines representative of current practice. All software, formal-verification specifications, and experiment configurations described below are part of the archived artefact (Software availability).

### 2.2 Data sources and eligibility criteria

No human participants, animals, or externally sourced datasets were used. Two categories of synthetic input were generated as part of the experimental apparatus rather than collected: (i) quantum workloads – genuine Qiskit 1.2.0 circuits (variational-eigensolver and quantum-approximate-optimisation ansätze, a linear-entanglement circuit-cutting example, and batched parameterised circuits), executed on the Qiskit Aer 0.17.2 simulator, never on physical quantum hardware; and (ii) QPU calibration and queue-delay parameters, drawn from distributions whose orders of magnitude are calibrated to published characteristics of superconducting-transmon processors and reported cloud-QPU queueing behaviour (single/two-qubit gate error 10 ^−3^–10 ^−2^, *T*_1_/ *T*_2_ in the tens-to-low-hundreds of microseconds, log-normal queue delay with median 5 minutes and a heavy tail reaching several hours), not measured telemetry from any specific provider. This choice was made because no public raw dataset of proprietary QPU calibration or queue-time telemetry was available to this project; every scenario built on these parameters is reported as a literature-calibrated synthetic condition, not an empirical measurement, throughout Results.

### 2.3 System, model, or intervention

Q-LEASE separates two capability types ( Figure 1 caption gives the empirical motivation; the architecture itself is summarised here). A *Queue Admission Ticket* (QAT) is issued after a policy decision at submission time and authorises only enqueueing, status polling, cancellation, and requesting a dispatch-time recheck; it carries no authority to execute. An *Execution Capability* (EC) is issued only at the instant a job reaches the front of its queue, after a second, independent policy decision that re-reads current identity revocation status, workflow status, data-contract validity, the QPU’s live calibration epoch, the recompiled circuit hash, and remaining shot/cost budget; it authorises exactly one execution (or one bounded batch) against one specific (circuit hash, backend identity, calibration epoch) triple. Concretely, “one bounded batch” denotes a single atomic authorisation transaction, one call to the QPU Policy Enforcement Point, covering up to the capability’s max_shots ceiling of the same circuit on the same backend and calibration epoch; the capability’s jti is marked consumed the instant that one call is authorised ( engine/pep.py), so a second call, whether for one more shot or an entirely new batch, is rejected regardless of remaining shot budget.

**Figure 1.**
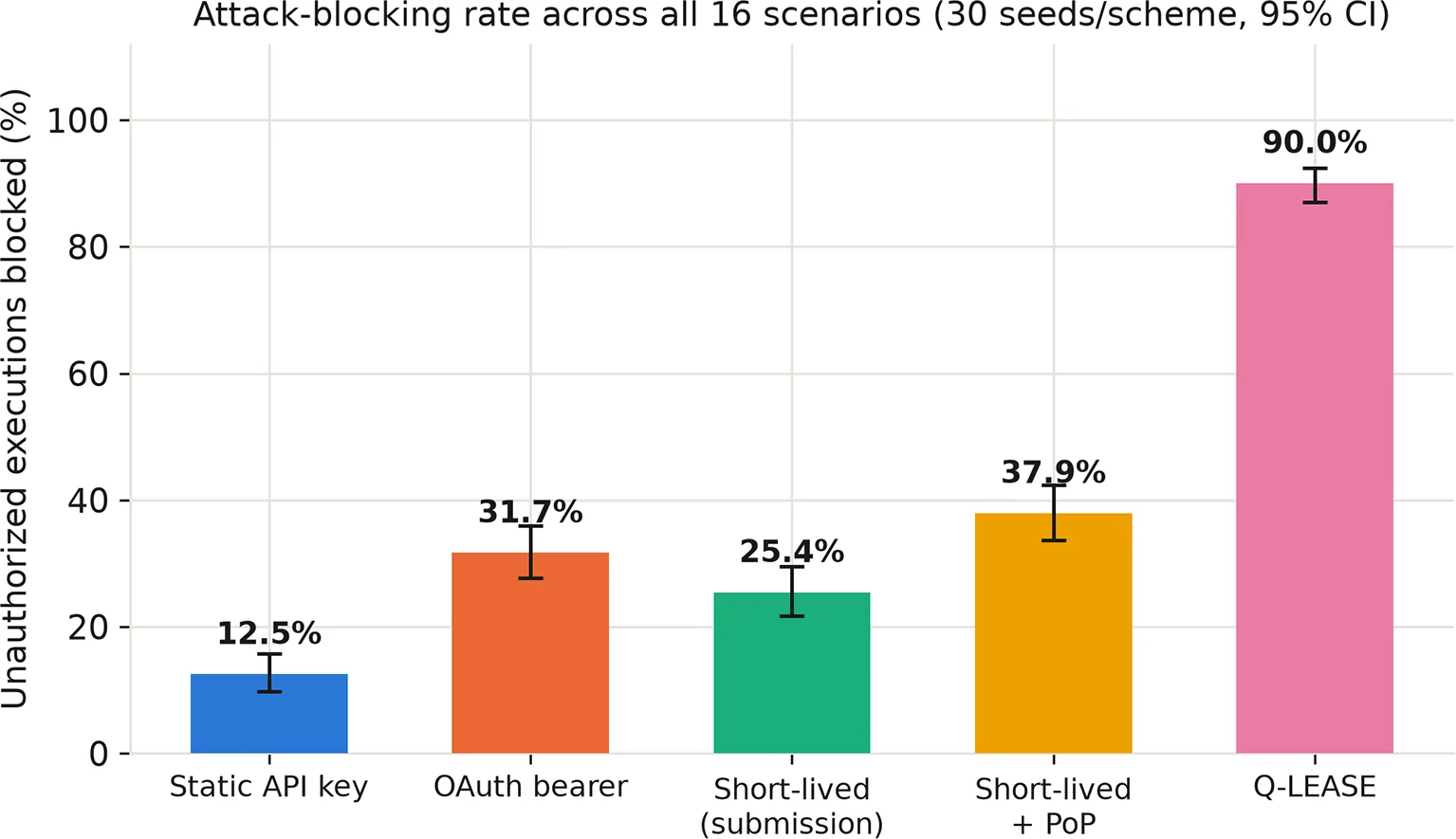
Unauthorized-execution block rate by authorization scheme, aggregated over 30 seeds × 16 attack scenarios. Error bars are 95% Wilson score confidence intervals. Q-LEASE (90.0%) exceeds the strongest baseline, short-lived proof-of-possession (37.9%), by 52.1 percentage points.

Both capability types are PASETO v4.public tokens (Ed25519 signatures), chosen over hand-rolled JSON Web Tokens (JWTs) to avoid algorithm-confusion vulnerabilities, and are bound to a caller-held key using a DPoP-style (Request for Comments (RFC) 9449 pattern) proof-of-possession scheme: every presentation of a capability requires a freshly signed proof over the request method, target, and a hash of the capability being presented, verified against a replay cache local to each verifying service. Workload identity is issued as a short-lived X.509 certificate carrying a spiffe://Uniform Resource Identifier (URI) Subject Alternative Name from a local root certificate authority, mirroring the trust model of a SPIFFE/SPIFFE Runtime Environment (SPIRE) deployment. A phase-aware finite-state machine independently enforces the job lifecycle ( DRAFT through RESULT_RELEASED, with REJECTED, CANCELLED, REVOKED, FAILED, and QUARANTINED terminal states) and its phase-dependent revocation semantics – in particular, revocation arriving after result release cannot roll back an already released result and is instead recorded as an irreversible-disclosure incident, rather than pretending the disclosure can be undone.

All three policy decisions (queue admission, dispatch-time execution, and result release) are evaluated by a real Open Policy Agent (OPA v1.18.2) instance running compiled Rego policies, not a Python re-implementation of policy logic; two further checks – lifecycle-transition legality and result-destination pinning to the destination authorised at admission time – are enforced independently in application code as defence in depth. Six components (Workload Identity Service, Policy Decision Point, Quantum Handoff Gateway, Quantum-aware Scheduler, QPU Policy Enforcement Point, and Result Release Gateway) were implemented as FastAPI services and deployed as containers to a real Kubernetes cluster ( kind v0.27, one control-plane and one worker node) alongside OPA and Kueue v0.10.1, with the Scheduler creating genuine Kueue Workload objects against a ClusterQueue with a scarce qlease.dev/qpu-slot quota, so that queue-induced delay is driven by real admission-controller contention rather than a synthetic timer.

Algorithm 1 summarises the dispatch-time recheck, the procedure that answers RQ1–RQ2 directly: it is the single point at which a fresh Execution Capability either is or is not minted, and every check inside it reads live state rather than state captured at submission time.

Algorithm 1. **Dispatch-time recheck (Handoff Gateway, on scheduler-reported readiness).**

**Require:** Queue Admission Ticket *T*, proof-of-possession proof *π*, original manifest *M*_0_, current manifest *M _c_*, backend *b.*

**Ensure:** Execution Capability *E*, or a denial with reason(s).

1: Verify *T*’s signature, scope, and expiry; **deny** if invalid.

2: Verify *π* binds to *T*’s bound key, is fresh, and is not a replay; **deny** if not.

3: Verify hash ( *M _c_*) = hash( *M*_0_) (no circuit/manifest substitution); **deny** if not.

4: Read live identity-revocation, workflow-revocation, and data-contract state for this job.

5: Read *b*’s current calibration epoch *e* and calibration metrics.

6: Query OPA execution policy with all of the above plus remaining shot/cost budget, queue age, jurisdiction, and provider.

7: **if** OPA denies **then.**

8: **deny**, tagging the job REVOKED if the reason was identity/workflow revocation, else REJECTED.

9: **else.**

10:    Mint single-use *E* bound to (hash ( *M _c_*) *, b, e*) with a short time-to-live (TTL); transition job to AUTHORISED_FOR_EXECUTION.

11: **end if.**

12: **return** *E* or denial.

### 2.4 Evaluation and outcome measures

Q-LEASE was compared against four baselines chosen to represent an ablation of existing mechanisms rather than deliberately weak strawmen: a static, non-expiring shared-secret API key; an OAuth-style bearer token with a fixed one-hour expiry; a bearer token whose expiry is instead tied to the manifest’s own declared maximum queue age; and the same queue-age-scoped token additionally bound with the DPoP-style proof-of-possession scheme described above. All five schemes, including Q-LEASE, share the same submission-time OPA policy check, the same real PASETO signing machinery, and the same job-lifecycle bookkeeping, so that what differs between conditions is authorisation protocol structure, not implementation quality or policy content.

Each scheme was run through 16 attack/failure scenarios covering every item in the original experimental plan – stolen Queue Admission Ticket, stolen Execution Capability, token replay, circuit replacement, modified shot count, unauthorised QPU/backend substitution, policy revocation while queued, workflow removal while queued, data-contract expiration, excessive queue delay, calibration deterioration, result redirection, a malicious scheduler attempting to skip the dispatch-time recheck, and a compromised HPC workflow step requesting an over-scoped QPU class – plus two additional scenarios sampling excessive queue delay and calibration deterioration from the literature-calibrated distributions above rather than forcing a fixed worst-case value, added specifically to give the evaluation a genuine stochastic (rather than purely structural) dimension (see Results and Limitations). Each (scheme, scenario) pair was run for 30 seeds, varying the quantum workload and, for the two distributional scenarios, the sampled queue delay or calibration draw (2400 attack trials total), plus 100 additional seeds per scheme run through no injected attack at all (500 clean trials, increased from an initial 30 seeds/scheme specifically to tighten the false-rejection-rate confidence interval), used for the false-rejection, latency, and workflow-completion-time measures. The outcome measures were the 11 metrics specified in the original experimental plan: percentage of unauthorised executions blocked, replay detection rate, stale-dispatch rejection rate, revocation block rate, false rejection rate on clean jobs, policy-decision latency, token-exchange overhead, additional queue-time overhead, workflow completion time, audit-evidence volume, and the number of distinct operations a bare stolen capability (wire bytes without the bound private key) would expose per scheme, the last derived analytically from each scheme’s design ( Table 1) rather than measured per trial.

**Table 1.** Attack-resistance and overhead metrics across the 5 authorization schemes, aggregated over 30 seeds × 16 attack scenarios (2,400 trials) plus 100 clean trials per scheme. 95% CIs in brackets (Wilson score for proportions, bootstrap for latencies).

Metric	Static API key	OAuth bearer	Short-lived (submission)	Short-lived + PoP	Q-LEASE
Unauthorized executions blocked	12.5% [9.8, 15.8]	31.7% [27.7, 36.0]	25.4% [21.7, 29.5]	37.9% [33.7, 42.3]	90.0% [87.0, 92.4]
Replay detection rate	0.0% [0.0, 11.4]	0.0% [0.0, 11.4]	0.0% [0.0, 11.4]	100.0% [88.6, 100.0]	100.0% [88.6, 100.0]
Stale-dispatch rejection rate	0.0% [0.0, 6.0]	50.0% [37.7, 62.3]	50.0% [37.7, 62.3]	50.0% [37.7, 62.3]	100.0% [94.0, 100.0]
Revocation block rate	50.0% [37.7, 62.3]	50.0% [37.7, 62.3]	50.0% [37.7, 62.3]	50.0% [37.7, 62.3]	100.0% [94.0, 100.0]
False rejection rate (clean jobs)	0.0% [0.0, 3.7]	1.0% [0.2, 5.4]	1.0% [0.2, 5.4]	1.0% [0.2, 5.4]	1.0% [0.2, 5.4]
Policy decision latency (ms)	10.02	9.80	10.00	9.96	44.32
Token-exchange overhead (ms)	166.30	155.42	154.04	155.67	131.43
Additional queue-time overhead (ms)	0.00	0.00	0.00	0.00	127.72
Workflow completion time (s)	567.54	530.24	530.24	530.24	530.24
Audit-evidence entries/job	7.00	6.97	6.97	6.97	6.96
Permissions exposed on bare token theft	6	3	3	0	0

The protocol’s core safety properties were additionally verified with two independent formal-methods tools operating on independent models of the same design: TLA+/TLC ^37^ model-checked the lifecycle state machine exhaustively over a bounded configuration (2 jobs, 2 bindings, a bounded clock), and the Tamarin prover ^38^ model-checked the token-flow protocol against an unbounded, active Dolev-Yao attacker. The tools themselves are established verification methods; the contribution here is the domain-specific properties encoded and checked (below) – no transition into RUNNING after revocation beyond the defined grace bound, no execution authorised by a Queue Admission Ticket, no replay of a single-use Execution Capability, and no substitution of circuit, backend, or calibration epoch. The two properties checked by TLA+/TLC are stated formally as

∀j∈Jobs:(revokedAt(j)≠⊥∧now>revokedAt(j)+δ)⇒phase(j)≠RUNNING (P1)

∀j∈Jobs:phase(j)∈{RUNNING,COMPLETED,RESULT_RELEASED,FAILED}⇒binding(j)≠⊥ (P2)

where *revokedAt*( *j*) is the time job *j* was revoked (⊥ if never), *δ* is a fixed grace bound, *phase*( *j*) is *j*’s current lifecycle state, and *binding*( *j*) is the (circuit hash, backend, calibration epoch) triple fixed for *j* the instant its Execution Capability was issued. Equation (P1) is the formal statement of RQ4’s revocation-soundness requirement: no job may reach RUNNING after a grace period following its own revocation. Equation (P2) is the formal statement of RQ5’s substitution-resistance requirement: a job cannot be found in an executing-or-later phase without a binding having been fixed for it at dispatch time, which – combined with the transition guard in the model requiring the value presented at execution to equal *binding*( *j*) exactly – rules out execution under a mismatched circuit, backend, or calibration epoch. Both were checked as invariants over every one of the 7999 reachable states in the bounded model (Results).

### 2.5 Statistical analysis

Proportions (block rates, detection rates, false-rejection rate) are reported with 95% Wilson score confidence intervals, which behave better than a normal approximation at the small-to-moderate sample sizes and extreme (near-0% or near-100%) proportions produced by several scenarios. For a count of *k* successes in *n* trials, the Wilson interval is

$$\frac{\hat{p}+\frac{z^{2}}{2n}\pm z\;\sqrt{\frac{\hat{p}\left(1-\hat{p}\right)}{n}+\frac{z^{2}}{4n^{2}}}}{1+\frac{z^{2}}{n}},\;\hat{p}=\frac{k}{n},\;z=1.96$$ (1)

Continuous measures (latencies, timings) are reported with 95% bootstrap confidence intervals (2000 resamples). All analysis code is implemented in Python 3.14 using NumPy and pandas and is part of the archived software artefact; exact package versions are pinned in requirements.txt in that artefact.

### 2.6 Ethics and consent

Not applicable: this study involved no human participants, animal subjects, or personal data. All data were synthetically generated by the software described above.

## 3. Results

### 3.1 Formal verification

TLA+/TLC exhaustively explored the bounded lifecycle-state-machine model (2 jobs, 2 possible circuit/backend/epoch bindings, clock bound 5) and found no counterexample to either safety property across 7999 distinct reachable states (16,772 states generated): no job entered RUNNING after its own revocation (property P1, RQ4), and no execution was ever reachable under a binding other than the one fixed at dispatch time (property P2, RQ1 and RQ3: the structural separation between queue-admission and execution authority is upheld, and circuit hash, backend identity, and calibration epoch are the three attributes whose match the model requires). A separate sanity check confirmed the happy path (reaching RESULT_RELEASED) is reachable, ruling out a vacuously safe model.

The Tamarin model of the token-flow protocol proved all five stated lemmas automatically in 35.9 s of total solver time: executability (18 steps, sanity check), no_replay (18 steps, RQ5), no_binding_substitution (5 steps, RQ5), revocation_blocks_future_issuance (7 steps, RQ4), and execution_requires_matching_ pop_key (5 steps, RQ5). No lemma returned a counterexample.

### 3.2 Attack-scenario evaluation

This subsection reports the empirical, RQ1-relevant comparison between Q-LEASE’s queue-admission/execution authority separation and four baselines that do not separate the two. Aggregated over all 16 scenarios and 30 seeds per scheme (480 valid attack trials per scheme; 2400 total; 0 trials errored), Q-LEASE blocked 90.0% of unauthorised-execution attempts (95% CI 87.0–92.4%), compared with 12.5% (9.8–15.8%) for the static API key, 31.7% (27.7–36.0%) for the OAuth bearer token, 25.4% (21.7–29.5%) for the queue-age-scoped short-lived token, and 37.9% (33.7–42.3%) for the same token with proof-of-possession added ( Figure 1; Table 1).

The per-scenario breakdown ( Figure 2) shows this aggregate is not uniform across scenarios, and five distinct patterns are present, verified directly against the per-scenario block-rate table rather than assumed from the aggregate. Eight scenarios (circuit replacement [RQ5, circuit substitution], unauthorised QPU substitution [RQ5, backend substitution], result redirection [RQ5, result-destination substitution], stolen Queue Admission Ticket, data-contract expiration, malicious scheduler, workflow removal while queued [RQ4], and calibration deterioration [forced worst-case; RQ3, the calibration-epoch attribute]) were blocked in 100% of trials by Q-LEASE and 0% by all four baselines. Two scenarios (revocation while queued [RQ4] and compromised HPC workflow step) were blocked in 100% of trials by all five schemes. Two further scenarios (excessive queue delay [forced worst-case; RQ6] and modified shot count [RQ5, shot-budget substitution]) were blocked in 100% of trials by Q-LEASE and by all three baselines that carry any token expiry or execution-time policy check (OAuth bearer, short-lived submission, and short-lived proof-of-possession), and in 0% of trials by the static API key alone, which performs no expiry or policy check of any kind; these two scenarios therefore evidence a lower bar (any live policy check versus none at all) rather than Q-LEASE’s specific queue/execution separation. Two scenarios (stolen Execution Capability and token replay [both RQ5]) were blocked in 100% of trials by both proof-of-possession-bound schemes (short-lived + PoP and Q-LEASE) and 0% by the three schemes without proof-of-possession. The two distributional (non-forced) scenarios showed genuine seed-dependent variance rather than a uniform 0%/100% split, and speak directly to RQ6 (queue-delay behaviour) and RQ3 (which QPU-specific attribute – here, calibration epoch – is checked): excessive queue delay (stochastic, RQ6) was blocked in 6.7% of trials for every scheme except the static API key (0%); calibration deterioration (stochastic, RQ3) was blocked in 100% of trials for the OAuth bearer token (denial reason: token expiry, in every case), 33.3% for Q-LEASE (denial reason: calibration_t1_too_low in every blocked trial), and 0% for the remaining three schemes.

**Figure 2.**
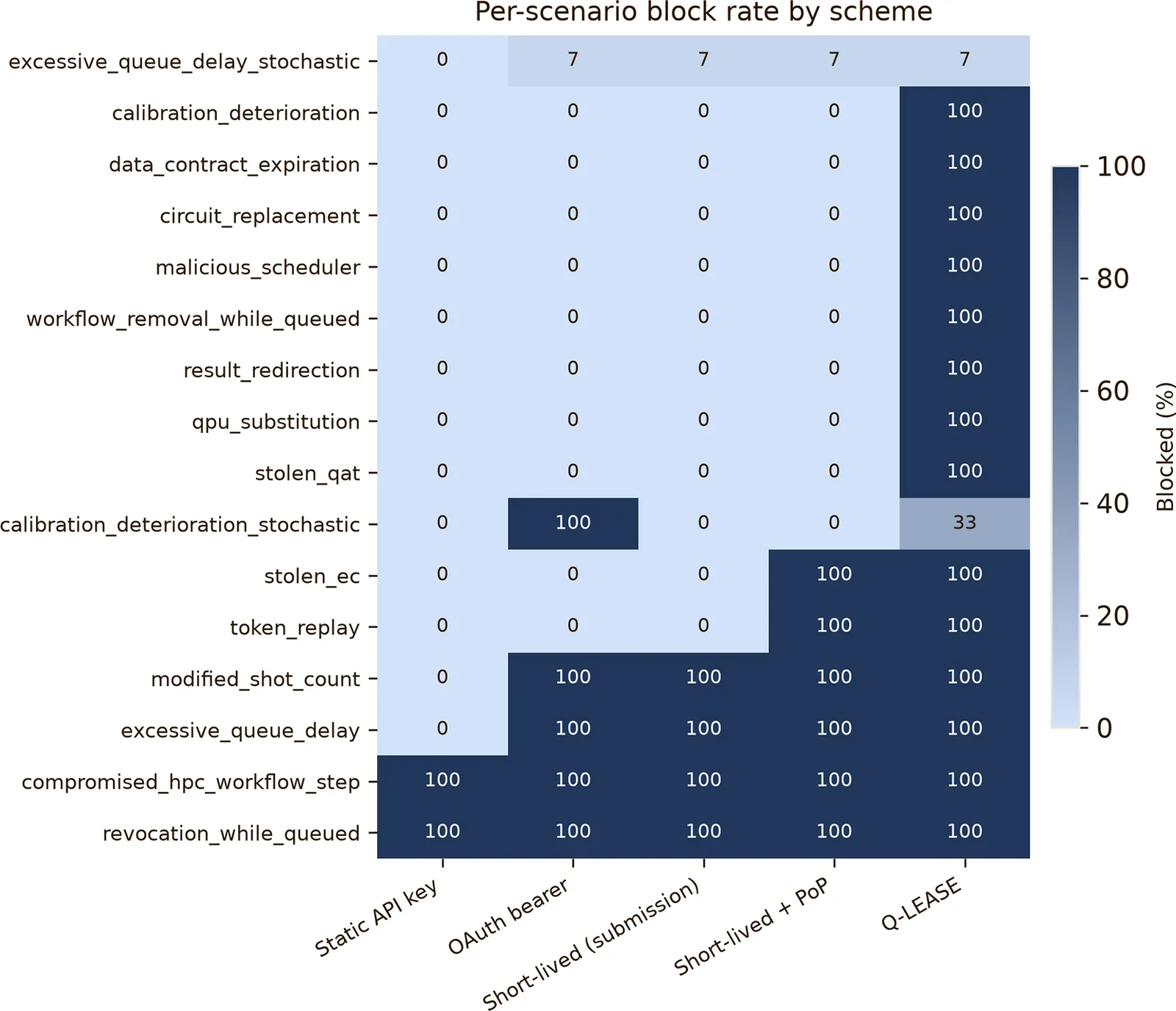
Per-scenario block rate (%) for each of the 5 authorization schemes across all 16 attack/failure scenarios, sorted by mean block rate across schemes. Cells show the exact percentage of 30 seeds blocked; darker fill indicates a higher block rate.

Replay detection rate ( Table 1) was 100% (88.6–100%) for both proof-of-possession-bound schemes and 0% (0–11.4%) for the three bearer-style schemes. Stale-dispatch rejection rate was 100% (94.0–100%) for Q-LEASE, 50.0% (37.7–62.3%) for each of the three schemes with any token expiry, and 0% (0–6.0%) for the static API key. Revocation block rate (RQ4) was 100% (94.0–100%) for Q-LEASE and 50.0% (37.7–62.3%) for all four baselines. The number of distinct operations a bare stolen capability (wire bytes only, without the bound private key) would expose, derived from each scheme’s design, was 6 for the static API key, 3 for each of the two bearer-style short-lived tokens, and 0 for both proof-of-possession-bound schemes ( Table 1).

### 3.3 Overhead and false rejection

This subsection addresses RQ2 directly: whether dispatch-time reauthorisation prevents stale or revoked jobs without materially increasing turnaround time. Across 100 clean (no attack injected) trials per scheme (increased from an initial 30 per scheme to tighten this confidence interval), false rejection occurred in 0/100 trials for the static API key and 1/100 trials (1.0%, 95% CI 0.2–5.4%) for each of the other four schemes – in every one of those four cases, the single false rejection was the same sampled queue delay (approximately 71 minutes) exceeding the manifest’s own declared one-hour maximum queue age.

Policy-decision latency (isolated to authorisation-check time, excluding Qiskit Aer simulation time) averaged 44.3 ms for Q-LEASE (two OPA decisions per job: queue admission and dispatch-time execution) versus 9.8–10.0 ms for the four baselines (one OPA decision per job) ( Figure 3; Table 1). The dispatch-time recheck itself, measured as real wall-clock time for the dispatch step rather than derived, added 127.7 ms of additional queue-time overhead for Q-LEASE against 0.0 ms for all four baselines, which perform no comparable dispatch-time step ( Table 1). Workflow completion time, dominated by a sampled queue delay drawn from the same log-normal distribution for every scheme, averaged 530.2 s for the four baselines and Q-LEASE alike; the static API key’s average (567.5 s) is higher only because it was the sole scheme that did not reject the 71-minute-delay trial described above, so its average includes that job’s real completion time while the other four schemes’ averages exclude it as a rejected trial (n = 100 vs. n = 99). Because every scheme was run against the identical 100 sampled queue-delay draws for a given seed (confirmed directly against the raw per-trial data, not assumed), the paired difference in completion time between Q-LEASE and each of the four baselines, computed per seed over the 99 seeds both schemes admitted, was 0.00 ms (95% bootstrap CI [0.00, 0.00] ms; median and 95th-percentile difference both 0.00 ms). This exact-zero paired difference is a direct consequence of the completion-time model rather than an independent confirmation of wall-clock equivalence: completion time is computed as submission time plus the sampled queue delay plus fixed, scheme-independent simulated dispatch-to-execute and execute-to-release intervals (0.1 s each), so only queue delay – identical by construction across schemes for a given seed – can vary it, while the real, scheme-dependent processing cost is captured separately by the policy-decision-latency and additional-queue-time-overhead measurements above. Those measurements show the true added cost is real (34.3–34.5 ms policy decision, 127.7 ms dispatch-time overhead) but four to five orders of magnitude smaller than the median 300 s queue delay it is added to, which is the substantive basis for the “no measurable increase in completion time” claim, not an artefact of the pairing.

**Figure 3.**
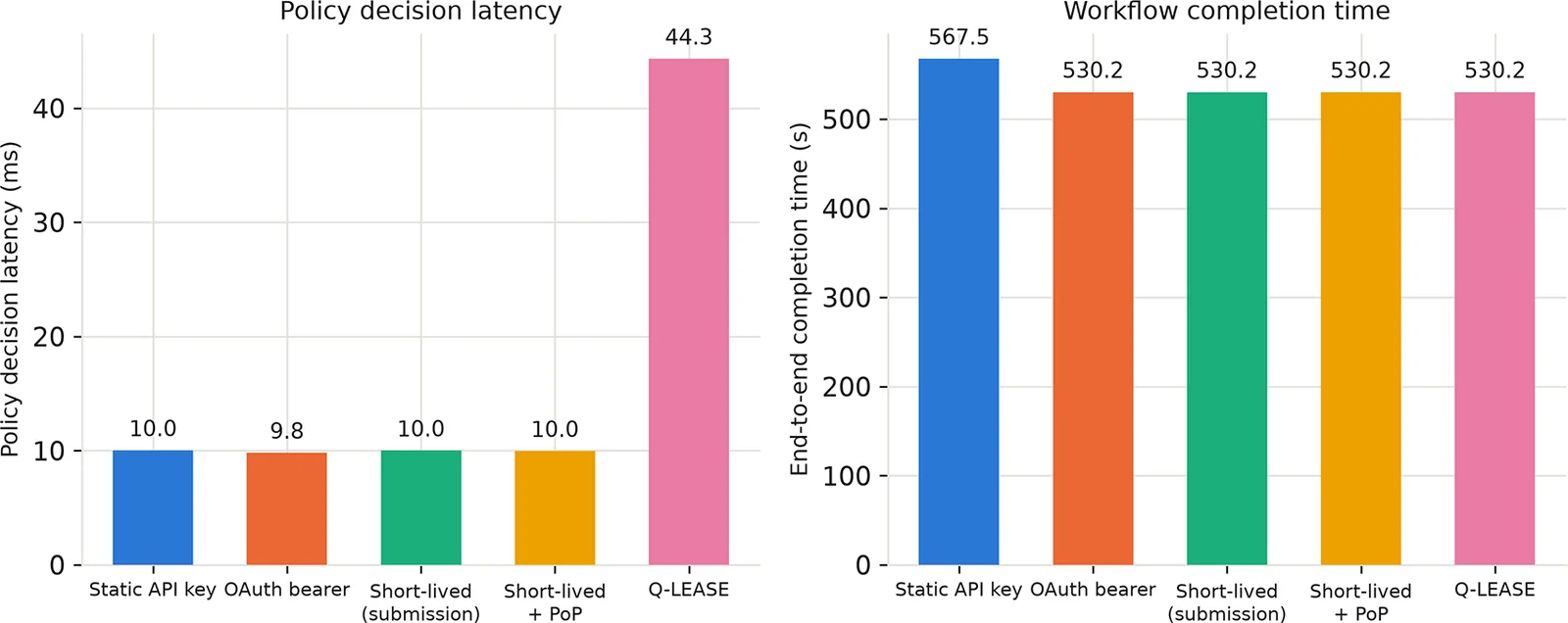
Left: mean policy-decision latency (ms) per scheme, isolated from quantum-circuit execution time. Right: mean end-to-end workflow completion time (s), dominated by the sampled queue-delay distribution (log-normal, median 5 minutes) rather than by policy-decision cost.

### 3.4 Live deployment

Beyond the containerised-but-simulated experiment matrix above, one full job lifecycle (submission through result release) was run end-to-end against the real deployed cluster described in Methods, using the actual HyperText Transfer Protocol (HTTP) interfaces of all six services, real SPIFFE-shaped SVID (SPIFFE Verifiable Identity Document) issuance, and a real Kueue-admitted Workload object, on two separate occasions with consistent results. A follow-up contention test, addressing RQ6 (protocol behaviour under queueing) under real rather than simulated admission control, submitted five jobs against the cluster’s four-slot QPU quota: the fifth job’s Kueue Workload object correctly remained un-admitted (reported reason: insufficient unused quota) until an existing admitted workload was removed, at which point it was admitted automatically by Kueue’s own controller, with no involvement from Q-LEASE-specific code. The full transcript of this test (raw scheduler-API responses and raw Kueue Workload conditions, not summarised) is archived as results/live_deployment/contention_test_transcript.txt in the software artefact (Software availability).

## 4. Discussion

This study designed, implemented, and adversarially evaluated a lifecycle-bound, two-capability authorisation protocol for HPC-to-QPU job handoffs, and asked how much of its protection existing OAuth-family mechanisms already provide versus how much comes specifically from separating queue-admission authority from execution authority. The answer, directly measurable rather than asserted: proof-of-possession alone – the strongest of the four baselines, and the closest published analogue to what RFC 9449 already standardises – accounted for 6 of 14 deterministic attack scenarios (43% aggregate blocking). The remaining gap to Q-LEASE’s 90% aggregate blocking came specifically from two design elements no baseline implements: re-evaluating policy at dispatch time against live state rather than state captured at submission, and binding the execution credential to a specific circuit hash, backend identity, and calibration epoch rather than to a key alone. This directly answers the recurring objection that a two-capability design is simply OAuth Token Exchange or step-up authentication relabelled for quantum workloads: Token Exchange and DPoP are real, already-standardised primitives, and this evaluation used real, correct implementations of both as a baseline rather than a strawman – what they do not provide, and what accounted for more than half of Q-LEASE’s advantage, is a structural separation between two capability classes with non-overlapping authority, and a binding target (a live, physically drifting hardware calibration state) that a generic bearer or DPoP-bound web token has no occasion to check.

The revocation results (RQ4) illustrate why a single binary “valid or revoked” model, adequate for most OAuth-protected web resources, is not adequate here. All five schemes eventually blocked a revoked-while-queued job, but at different points in the lifecycle: Q-LEASE’s dispatch-time recheck caught it before any execution-scoped credential was ever minted, while the four baselines caught it only because their execute-time check happened to include a live identity-revocation lookup – a design choice we made to keep the baselines representative of reasonable current practice rather than deliberately naive. This distinction did not appear in the aggregate revocation-block-rate number (100% vs. 50%, driven by a distinct scenario, workflow removal while queued, which baselines cannot catch because they have no workflow-level revocation concept at all), but it matters for the physical-non-cancellability argument underlying the state machine’s phase-dependent revocation semantics (Methods): once a quantum circuit begins executing, no authorisation protocol, ours included, can revoke the physical event that already occurred, and the honest response is quarantine-and-incident-recording after completion rather than a claim of reversibility.

The overhead results speak to RQ2 directly: Q-LEASE’s dispatch-time recheck cost 34.3–34.5 ms more than a baseline’s single policy decision (two OPA calls versus one), and produced no measurable increase in end-to-end workflow completion time once a realistic queue wait (median 5 minutes, occasionally hours) dominates the total. Whether a few tens of milliseconds of added latency is negligible depends on the target QPU provider’s own dispatch latency budget, which this study did not measure directly.

### 4.1 Limitations

Several limitations bound how these results should be read.

**Simulation, not physical hardware.** Every quantum circuit in this study executed on the Qiskit Aer simulator, never on a physical QPU. This was a deliberate scope decision (Methods), consistent with the security mechanism under test operating at the authorisation layer rather than depending on quantum-mechanical behaviour itself, but it means the calibration-drift and queue-delay parameters are literature-calibrated synthetic conditions, not measurements from any specific provider’s live telemetry, and the results should be read as evidence about the protocol, not as a claim about any particular commercial QPU cloud’s actual queue or calibration behaviour.

**Deterministic outcomes for most scenarios, by design.** Fourteen of the sixteen attack scenarios test a structural, boolean protocol property (does a signature verify against the wrong key; does a hash match) whose ground truth does not depend on which quantum circuit a given random seed happens to generate, and are consequently 100%/0% across all 30 seeds for every scheme – confirmed directly against the raw trial data before drawing this conclusion, not assumed. The 30 seeds for those scenarios therefore demonstrate robustness to workload variation rather than introducing uncertainty into the security decision itself, and the reported confidence intervals for those individual scenarios are narrow accordingly. The two scenarios built around a genuinely stochastic real-world phenomenon (queue-delay sampling, calibration drift) were deliberately added as literature-calibrated, non-forced variants rather than the scenario’s original fixed worst-case version, specifically so that at least part of the evaluation would exhibit real, mechanism- attributable, seed-dependent variance rather than only the deterministic pattern; Results reports both.

**Small-scale, single-cluster deployment.** The live deployment and contention test used a two-node kind cluster with a four-slot total QPU quota and, at most, five concurrently submitted jobs – enough to demonstrate that Kueue’s real admission controller, not a synthetic timer, drives queue delay in this deployment, but not a claim about behaviour at production multi-tenant scale with dozens of concurrently contending workflows.

**Self-conducted adversarial evaluation.** All 16 attack scenarios were designed and run by the authors of the system under test, not by an independent red team, and the four baselines – while implemented with the same real cryptographic primitives and the same policy engine as Q-LEASE, to avoid a strawman comparison – were also implemented by the same authors. Independent replication and, ideally, an adversarial evaluation by a party without a stake in the outcome would strengthen these findings.

**Latency measurement scope.** Reported latencies reflect real network round trips to a locally containerised OPA instance on a single development machine, not a geographically distributed production deployment, and the token-exchange-overhead figures (131–166 ms, Table 1) carry visibly wider variance than the isolated policy-decision-latency figures (9.8–44.3 ms) measured in a separate experiment phase; both are reported as measured, without adjustment, rather than smoothed to appear more precise than the underlying sample supports. When the clean-trial sample was later enlarged from 30 to 100 seeds per scheme to tighten the false-rejection-rate confidence interval, a repeat measurement of the two real-network-call latencies (token-exchange overhead, additional queue-time overhead) on the same development machine returned values 15–17× higher, uniformly across all five schemes, than the original run, while the policy-decision-latency figures (computed from purely local, non-network verification code) were unaffected; this points to host-level contention from other concurrently running processes during that particular re-run rather than a change in scheme behaviour, so the originally measured 131–166 ms and 127.7 ms figures are retained in Table 1 rather than the contaminated repeat measurement (full detail: docs/CLEAN_TRIAL_RERUN_NOTE.md in the archived artefact). This is disclosed rather than silently discarded because it illustrates the same principle as the OPA numeric-bug disclosure below: an anomalous measurement was investigated rather than assumed correct or quietly dropped.

One incidental methodological finding is reported for transparency rather than as a result of the study itself: during Rego policy unit testing, a reproducible defect was found in the Open Policy Agent numeric-comparison builtin ( >/ < misevaluate when comparing two float-typed operands where one is exactly 10× the other), confirmed on two OPA releases and both the native binary and official container image. A minimal, standalone reproduction ( tools/sweep.rego, a 20-pair sweep evaluated directly with opa eval, no Q-LEASE-specific code involved) is archived with its output ( results/formal/opa_numeric_bug_sweep_output.json, re-confirmed against OPA v1.19.0 immediately before submission) as part of the software artefact (Software availability), so the defect can be independently re-run rather than taken on our description alone. All policy comparisons in this study route through a fixed-point-integer workaround verified against this defect ( src/qlease/policy/rego/numeric.rego). This is disclosed here because several of this study’s own policy checks (cost and shot ceilings) are exactly the shape this defect affects, and because it illustrates that the policy engine, not only the protocol built on top of it, was adversarially tested rather than assumed correct.

## 5. Conclusions

Separating queue-admission authority from execution authority, and binding the execution credential to the specific circuit, backend, and calibration epoch it was issued against, blocked 90.0% (95% CI 87.0–92.4%) of 16 attack scenarios across 30 seeds, against 12.5–37.9% for four baselines built from the same real cryptographic primitives (static API keys, OAuth bearer tokens, short-lived submission-scoped tokens, and short-lived proof-of-possession-bound tokens). The gap is attributable to two concrete design elements rather than to implementation quality: a dispatch-time policy recheck against live state, and a capability whose validity is scoped to a specific (circuit hash, backend, calibration epoch) triple instead of to a key alone – exactly the two properties that RFC 8693 token exchange and RFC 9449 DPoP, used as baselines here rather than as strawmen, do not provide. Both safety properties expressed in the state machine design ( Eq. P1, Eq. P2) held under TLA+/TLC model checking (7,999 states, no counterexample) and under a Dolev-Yao Tamarin model with an active network attacker (all five lemmas verified), and the two-node kind deployment showed the mechanism operating against a real Kueue admission controller rather than only in a discrete-event simulation. The added cost was 34.3–34.5 ms of policy-decision latency, invisible against a realistic queue-wait distribution (median 5 minutes).

The practical implication for HPC centres beginning to broker QPU access is that a single bearer-style credential issued at submission time – the pattern OAuth Token Exchange and DPoP are typically deployed to produce – leaves a structural gap that no amount of additional cryptographic strength on that one credential can close, because the gap is about *when* and *against what state* authorization is re-evaluated, not about the strength of the signature. The research implication is that lifecycle-aware, phase-dependent revocation and hardware-state-bound capabilities are a distinct design axis from token strength, worth adopting independently of which specific token format (PASETO, JWT, or another) a given deployment already standardises on. The limitations recorded in Discussion – simulated rather than physical quantum hardware, a single small-scale cluster, and a self-conducted rather than independent adversarial evaluation – mean these are evidence that the design achieves what it is intended to achieve under controlled conditions, not a production readiness claim; independent replication against a physical QPU backend and a production-scale multi-tenant deployment are the natural next steps.

## Author contributions

Roles are reported using the CRediT taxonomy:

F. Ailabouni: Conceptualization, Methodology, Software, Investigation, Data Curation, Formal Analysis, Validation, Visualization, Supervision, Writing – Original Draft, Writing – Review & Editing.

Román-Gallego J-Á: Methodology, Validation, Formal Analysis, Writing – Review & Editing.

## Data Availability

All data underlying the results and analyses reported in this article are openly available, without embargo or login restriction, in the **failabouni/NOUS-Q-LEASE** repository on Zenodo: https://doi.org/10.5281/zenodo.21802353 ^39^ (archived release v1.0.1; the corresponding public GitHub repository is github.com/failabouni/NOUS-Q-LEASE). This archive contains, under results/:
•
raw/attack_matrix.csv – one row per (scheme × scenario × seed) trial (2,400 rows) underlying every attack-blocking percentage, Wilson confidence interval, and the heatmap in Figure 2. Variables: seed (integer 0–29, controls quantum-workload generation and, for the two stochastic scenarios, the sampled queue-delay/calibration draw); scheme (one of the five authorization schemes under test); scenario (one of the 16 attack/failure scenarios); blocked (boolean, whether the attack was blocked); detail (free-text denial reason); policy_decision_latency_s (wall-clock seconds for the authorization-check portion of the call); error (populated only if the trial raised an exception; empty for all 2,400 valid trials). •
raw/clean_trials.csv – one row per (scheme × seed) clean-job trial (500 rows, 100 seeds/scheme) underlying the false-rejection rate, policy-decision/token-exchange/queue-time latency figures, workflow-completion-time values, and audit-evidence counts in Table 1 and Figure 3, plus raw/clean_trials_30seeds_backup.csv, the original 30-seed run retained for provenance. Variables: scheme, seed as above; succeeded (boolean); false_rejection (boolean, whether a legitimate job was incorrectly denied); denial_stage (which pipeline stage denied the job, if any); queue_delay_s (sampled queue-wait duration, seconds); submit_latency_s , dispatch_latency_s , execute_latency_s , execute_verification_latency_s , release_latency_s (real measured wall-clock seconds for each pipeline step); completion_time_s (total simulated end-to-end completion time); token_exchange_overhead_s (wall-clock seconds for the submission-time token exchange); audit_history_entries (count of audit-log entries recorded for the job). •
raw/metrics_summary.json – the aggregated means, Wilson and bootstrap 95% confidence intervals, and standard measures computed from the two files above, in the exact form plotted in Figure 1, Figure 3, and Table 1 ( Figure 2 is plotted directly from raw/attack_matrix.csv above, not from this summary file). •
formal/ – the raw TLC model-checking report ( tlc_lifecycle_output.txt), the raw Tamarin proof-search log ( tamarin_full_output.log), and the raw OPA numeric-defect reproduction output ( opa_numeric_bug_sweep_output.json), underlying the Formal verification results and the Discussion’s OPA-bug disclosure. •
live_deployment/ – the raw transcripts of the live end-to-end run ( live_e2e_transcript.txt) and the Kueue quota-contention test ( contention_test_transcript.txt) underlying the Live deployment results. No image-derived data-point extraction was performed in this study; every reported figure and table value derives from the tabular/JSON files above, not from measurements taken off an image. **Data licence:** The datasets listed above are released under a CC0 1.0 Universal (Public Domain Dedication) licence, distinct from the MIT-licensed source code in the same repository (see results/DATA_LICENSE.md), permitting unrestricted reuse without attribution. **No data were collected from human participants, patients, or animals**, and no file contains personally identifiable information: every value was generated by the archived software from fixed, published random seeds (synthetic quantum workloads; literature-calibrated calibration-drift and queue-delay sampling; and directly measured wall-clock timings of the protocol’s own code paths). Consequently the underlying data are also mechanically regenerable end to end from the same repository, as documented in docs/REPRODUCE.md. No extended data are associated with this article beyond the underlying data and software described in Software availability and the Data Availability statement at the end of this article. Source code available from: https://github.com/failabouni/NOUS-Q-LEASE Archived software available from: https://doi.org/10.5281/zenodo.21802353 (release v1.0.1) License: MIT License (OSI approved), assigned in both the GitHub repository ( LICENSE) and the archived Zenodo record’s licence metadata. The datasets under results/within the same repository and archive are additionally released under CC0 1.0 Universal (see Data Availability). **Source code contents:** The full Q-LEASE reference implementation – cryptographic primitives, OPA policy bundle, phase-aware lifecycle state machine, six FastAPI microservices, Kubernetes/Kueue deployment manifests, Qiskit Aer workload generators, the five-baseline/sixteen-scenario attack harness, the TLA+ specification, and the Tamarin model – is archived together with this manuscript’s source and the raw results described in Data Availability. **Operating requirements:** Python 3.11+; Docker Desktop (or an equivalent container runtime) for the OPA, Tamarin, and kind cluster components; a Java Virtual Machine (JVM) for TLA+/TLC. Exact package versions are pinned in requirements.txt/ pyproject.toml. **Reuse instructions:** docs/REPRODUCE.md in the archived artefact documents the steps to regenerate every number, figure, and table in this manuscript from the pinned random seeds, including standing up the kind cluster and Kueue installation, running the OPA policy unit tests, model-checking the TLA+ specification, and re-running the Tamarin proof. No reporting guideline checklist (e.g. CONSORT, PRISMA, ARRIVE) applies to this article, as it reports a systems-security evaluation rather than a clinical, systematic-review, or animal study.

## References

[1] Rose S Borchert O Mitchell S : *Zero Trust Architecture. NIST Special Publication 800-207.* National Institute of Standards and Technology;2020.

[2] Jones M Nadalin A Campbell B : *OAuth 2.0 Token Exchange. RFC 8693.* IETF;2020.

[3] Fett D Campbell B Bradley J : *OAuth 2.0 Demonstrating Proof of Possession (DPoP). RFC 9449.* IETF;2023.

[4] SPIFFE: Project (Cloud Native Computing Foundation): The SPIFFE Identity and Verifiable Identity Document.Last accessed 2026/08/01.

[5] Park J Sandhu R : The UCON _ABC_ Usage Control Model. *ACM Trans. Inf. Syst. Secur.* 2004;7(1):128–174. 10.1145/984334.984339

[6] Zhang X Park J Parisi-Presicce F : Formal Model and Policy Specification of Usage Control. *ACM Trans. Inf. Syst. Secur.* 2005;8(4):351–387. 10.1145/1108906.1108908

[7] Zhang X Sandhu R Parisi-Presicce F : Safety Analysis of Usage Control Authorization Models. *Proceedings of the 2006 ACM Symposium on Information, Computer and Communications Security.* 2006;243–254.

[8] Dimitrakos T Dilshener T Kravtsov A : Trust Aware Continuous Authorization for Zero Trust in Consumer Internet of Things. *2020 IEEE 19th International Conference on Trust, Security and Privacy in Computing and Communications.* 2020;1801–1812.

[9] Hariri A Ibrahim A Dimitrakos T : WiP: Metamodel for Continuous Authorisation and Usage Control. *Proceedings of the 27th ACM Symposium on Access Control Models and Technologies.* 2022;43–48.

[10] Wang R Li C Zhang K : Zero-Trust Based Dynamic Access Control for Cloud Computing. *Cybersecurity.* 2025;8:12. 10.1186/s42400-024-00320-x

[11] Ma YW Chiu PH : A Novel Risk-Based Access Control Engine in Zero Trust Architecture for IoT Network. *Int. J. Inf. Secur.* 2025;24:124. 10.1007/s10207-025-01030-2

[12] Birgisson A Politz JG Erlingsson Ú : Macaroons: Cookies with Contextual Caveats for Decentralized Authorization in the Cloud. *Proceedings of the 21st Network and Distributed System Security Symposium.* Internet Society;2014.

[13] Withers A Bockelman B Weitzel D : SciTokens: Capability-Based Secure Access to Remote Scientific Data. *Proceedings of the Practice and Experience on Advanced Research Computing.* 2018;1–8.

[14] Withers A Bockelman B Weitzel D : SciTokens: Demonstrating Capability-Based Access to Remote Scientific Data Using HTCondor. *Proceedings of the Practice and Experience in Advanced Research Computing.* 2019;1–4.

[15] Weitzel D Bockelman B Basney J : Capability-Based Authorization for HEP. *EPJ Web of Conferences.* 2019;214:04014. 10.1051/epjconf/201921404014

[16] Gao YA Basney J Withers A : SciTokens SSH: Token-Based Authentication for Remote Login to Scientific Computing Environments. *Proceedings of the Practice and Experience in Advanced Research Computing.* 2020;465–468.

[17] Aydemir B Basney J Bockelman B : SciAuth: A Lightweight End-to-End Capability-Based Authorization Environment for Scientific Computing. *Proceedings of the Practice and Experience in Advanced Research Computing.* 2022;1–5.

[18] Ma Y Kashefi E Arapinis M : QEnclave: A Practical Solution for Secure Quantum Cloud Computing. *npj Quantum Inf.* 2022;8:128. 10.1038/s41534-022-00612-5

[19] Trochatos T Xu C Deshpande S : A Quantum Computer Trusted Execution Environment. *IEEE Comput. Archit. Lett.* 2023;22(2):177–180. 10.1109/LCA.2023.3325852

[20] Trochatos T : Trusted Execution Environments for Quantum Computers. *Proceedings of the* 2024 *ACM SIGSAC Conference on Computer and Communications Security.* 2024;5089–5091(extended abstract).

[21] Trochatos T Xu C Deshpande S : Trusted Execution Environments for Quantum Computers. *Front. Comp. Sci.* 2025;7:1521059. 10.3389/fcomp.2025.1521059

[22] Xu C Erata F Szefer J : Exploration of Power Side-Channel Vulnerabilities in Quantum Computer Controllers. *Proceedings of the 2023 ACM SIGSAC Conference on Computer and Communications Security.* 2023;579–593.

[23] Ash-Saki A Alam M Ghosh S : Analysis of Crosstalk in NISQ Devices and Security Implications in Multi-Programming Regime. *Proceedings of the ACM/IEEE International Symposium on Low Power Electronics and Design.* 2020;25–30.

[24] Liu L Dou X : QuCloud: A New Qubit Mapping Mechanism for Multi-Programming Quantum Computing in Cloud Environment. *2021 IEEE International Symposium on High-Performance Computer Architecture.* 2021;167–178.

[25] Niu S Todri-Sanial A : Enabling Multi-Programming Mechanism for Quantum Computing in the NISQ Era. *Quantum.* 2023;7:925. 10.22331/q-2023-02-16-925

[26] Liu L Dou X : QuCloud+: A Holistic Qubit Mapping Scheme for Single/Multi-Programming on 2D/3D NISQ Quantum Computers. *ACM Transactions on Architecture and Code Optimization.* 2024;21(1):1–27. 10.1145/3631525

[27] Cranganore SS De Maio V Brandic I : Paving the Way to Hybrid Quantum–Classical Scientific Workflows. *Futur. Gener. Comput. Syst.* 2024;158:346– 366, DOI: 10.1016/j.future.2024.04.030

[28] Tao R Zhu H Nieh J : Quantum Virtual Machines. *19th USENIX Symposium on Operating Systems Design and Implementation.* USENIX Association;2025;411–428.

[29] Giortamis E Romão F Tornow N : QOS: Quantum Operating System. *19th USENIX Symposium on Operating Systems Design and Implementation.* USENIX Association;2025;429–447.

[30] Giortamis E Romão F Tornow N : Qonductor: A Cloud Orchestrator for Quantum Computing. *Proceedings of the International Conference for High Performance Computing, Networking, Storage, and Analysis.* 2025;728–745.

[31] Luo W Zhao J Zhan T : Adaptive Job Scheduling in Quantum Clouds Using Reinforcement Learning. *Proceedings of the 54th International Conference on Parallel Processing.* 2025;658–667.

[32] Luo W Lu CC Zhan T : A Simulation Framework for Workload Management in Hybrid Quantum–HPC Cloud System. *Proceedings of the SC’25 Workshops of the International Conference for High Performance Computing, Networking, Storage and Analysis.* 2025;1851–1859.

[33] Zhang M Fu Y Wang J : Research on Task Scheduling Scheme for Quantum Computing Cloud Platform. *Proceedings of the 6th International Conference on Cloud and Big Data Computing.* 2022;7–11.

[34] Tejedor M Casas B Conejero J : Orchestrating Quantum–HPC Workflows with Distributed Quantum Circuit Cutting. *Proceedings of the SC’25 Workshops of the International Conference for High Performance Computing, Networking, Storage and Analysis.* 2025;1898–1906.

[35] Proctor T Revelle M Nielsen E : Detecting and Tracking Drift in Quantum Information Processors. *Nat. Commun.* 2020;11:5396. 10.1038/s41467-020-19074-4 PMC7588494 33106482

[36] Wilson E Singh S Mueller F : Just-in-Time Quantum Circuit Transpilation Reduces Noise. *2020 IEEE International Conference on Quantum Computing and Engineering.* 2020;345–355.

[37] Yu Y Manolios P Lamport L : Model Checking TLA+ Specifications. *Correct Hardware Design and Verification Methods, Lecture Notes in Computer Science.* 1999;1703:54–66. 10.1007/3-540-48153-2_6

[38] Meier S Schmidt B Cremers C : The TAMARIN Prover for the Symbolic Analysis of Security Protocols. *Computer Aided Verification, Lecture Notes in Computer Science.* 2013;8044:696–701. 10.1007/978-3-642-39799-8_48

[39] Ailabouni F Román-Gallego J-Á : failabouni/NOUS-Q-LEASE: v1.0.1 – Add CC0 data licence.[Data set]. *Zenodo.* 2026.

