# Supplementary material for: Q-LEASE: Lifecycle-bound zero-trust authorization for HPC-to-QPU job handoffs
Source: Open Res Eur. 2026 Aug 24;6:302. [Version 1] doi: 10.12688/openreseurope.24782.1 (PMC13658992; doi:10.12688/openreseurope.24782.1)
Supplement: Peer review history

# Reviewer report: Reviewer response for version 1

**DOI:** 10.21956/openreseurope.26861.r81784

**Published:** 2026-10-12

**Authors:** Qasem Abu Al-Haija

**Affiliations:** 1 Jordan University of Science and Technology, Irbid, Irbid Governorate, Jordan

The manuscript presents an interesting approach to securing HPC-to-QPU workflows, supported by implementation and formal verification. However, several issues need further attention:

1. Novelty: Clearly explain what makes Q-LEASE different from existing authorization approaches.
2. Baselines: Include stronger comparisons to better demonstrate the benefits of the proposed method.
3. Security testing: Consider more realistic attacks, including compromised services, key theft, and concurrent requests.
4. Formal verification: Discuss the limitations of the verification models and consider possible race conditions.
5. Real-world validation: The experiments rely on simulated QPU workloads. Real hardware data would strengthen the findings.
6. Scalability: Evaluate the framework with more jobs, users, and distributed services.
7. Performance: Clarify the reported completion times and measure actual end-to-end overhead.
8. Statistical analysis: Interpret the 90% attack-blocking rate carefully, as most scenarios have deterministic outcomes.
9. Calibration results: Explain why only 33.3% of stochastic calibration-deterioration cases were blocked.
10. Distributed security: Clarify how token replay prevention and revocation work across multiple service instances.
11. Conclusions: Avoid broad practical claims that are not fully supported by the experiments.

Is the study design appropriate and does the work have academic merit?

Partly

Is the work clearly and accurately presented and does it cite the current literature?

Partly

If applicable, is the statistical analysis and its interpretation appropriate?

Partly

Are all the source data underlying the results available to ensure full reproducibility?

Partly

Are the conclusions drawn adequately supported by the results?

Partly

Are sufficient details of methods and analysis provided to allow replication by others?

Partly

Reviewer Expertise:

Cybersecurity, Network and Cloud Security, Zero-Trust Architecture, Access Control, Authentication and Authorization, IoT Security, AI-Driven Security, and Security Protocol Analysis. My expertise primarily covers the cybersecurity aspects of this work rather than quantum hardware implementation.

I confirm that I have read this submission and believe that I have an appropriate level of expertise to confirm that it is of an acceptable scientific standard, however I have significant reservations, as outlined above.

# Reviewer report: Reviewer response for version 1

**DOI:** 10.21956/openreseurope.26861.r81778

**Published:** 2026-10-09

**Authors:** Ilias cherkaoui

**Affiliations:** 1 South East Technological University, Waterford, Ireland

Q-LEASE solution manages to bring Zero-Trust authorization protocols to address the HPC-to-QPU gap. QAT alongside single-use EC before the check from OPA is able to deal with identity and calibration. The standard DPoP and OAuth with also Kubernetes containerization made it possible to perform attacks while still dealing with some residual latency of 44.3 ms. However, the scenarios were verifiable with TLA+/TLC and Tamarin making it up to 1% false rejection rate.

 As for the limitations and what might be addressed, some baselines for hardware-state binding in this case; also, it is worth mentioning that any claim here based on completion-time must be stated to be dependent on the model. Lastly, it would be fairly appropriate to dive into the details of as why 33.3% was the top reached for stochastic calibration.

Is the study design appropriate and does the work have academic merit?

Yes

Is the work clearly and accurately presented and does it cite the current literature?

Yes

If applicable, is the statistical analysis and its interpretation appropriate?

Yes

Are all the source data underlying the results available to ensure full reproducibility?

Yes

Are the conclusions drawn adequately supported by the results?

Yes

Are sufficient details of methods and analysis provided to allow replication by others?

Yes

Reviewer Expertise:

Quantum Cryptography, Zero-Trust AI, Quantum Networks and Protocols, Post-Quantum Cryptography, Nonlinear Dynamics, AI, Chaos Theory, Ergodic Number theory, Error correcting codes, Category theory.

I confirm that I have read this submission and believe that I have an appropriate level of expertise to confirm that it is of an acceptable scientific standard.

# Reviewer report: Reviewer response for version 1

**DOI:** 10.21956/openreseurope.26861.r80642

**Published:** 2026-09-15

**Authors:** Urooj Waheed

**Affiliations:** 1 DHA Suffa University, Karachi, Pakistan

The article presents a useful approach for authorization in HPC-to-QPU workflows. The problem is explained clearly, and the paper includes relevant work on Zero Trust, continuous authorization, capability-based access control, quantum security and scheduling. The methods are also given in enough detail. The authors have shared the source code, raw data, formal verification files and reproduction steps, so the work can be checked and repeated by other researchers.

 My main concern is the baseline comparison. Q-LEASE checks the policy again at dispatch time and also binds authorization with the circuit, backend and calibration state. The baseline methods do not include all these features. Because of this, it is not fully clear how much improvement comes from the two-capability design and how much comes from the extra dispatch-time check. An additional baseline or a simple ablation test would make this comparison stronger.

 The statistical results are useful, but some claims need more careful wording. The 90% attack-blocking result mostly comes from deterministic scenarios where the outcome is usually 0% or 100%. So this percentage and its confidence interval should be explained carefully. Also, the paper says there is no measurable increase in workflow completion time, but the same queue delays are used across the schemes and the real authorization overhead is measured separately. This point should be stated more clearly.

 The formal verification with TLA+/TLC and Tamarin is a strong part of the paper, but the experimental validation is still limited. The quantum workloads were run on Qiskit Aer and not on a physical QPU, and the live deployment was tested on a small two-node cluster. The authors already mention these limitations, but the conclusion should clearly say that the results are from controlled conditions and are not yet proof of production-scale performance. Overall, the paper has good academic value, but these points should be clarified before full approval.

Is the study design appropriate and does the work have academic merit?

Partly

Is the work clearly and accurately presented and does it cite the current literature?

Yes

If applicable, is the statistical analysis and its interpretation appropriate?

Partly

Are all the source data underlying the results available to ensure full reproducibility?

Yes

Are the conclusions drawn adequately supported by the results?

Yes

Are sufficient details of methods and analysis provided to allow replication by others?

Yes

Reviewer Expertise:

Network and IoT security, access control and authorization, blockchain-based security, and intelligent systems. My assessment mainly covers the security architecture, Zero-Trust/access-control design, methodology and evaluation. I have not assessed the quantum hardware aspects in depth.

I confirm that I have read this submission and believe that I have an appropriate level of expertise to confirm that it is of an acceptable scientific standard, however I have significant reservations, as outlined above.

## References

[1] : Trust Aware and Explainable Access Control for Internet of Medical Things: A Lightweight Hybrid Blockchain Approach. *IEEE Internet of Things Journal* .2026;13(12) : 10.1109/JIOT.2026.368009127843-27861 10.1109/JIOT.2026.3680091

[2] : Optimizing Smart-Home Energy Systems Through Energy-Efficient Off-Chain Blockchain-Based Attribute-Based Access Control (ABAC): A Hybrid LightGBM Approach. *Energies* .2026;19(10) : 10.3390/en19102279 10.3390/en19102279
